# Direct Observation of Sink-Dependent Defect Evolution in Nanocrystalline Iron under Irradiation

**DOI:** 10.1038/s41598-017-01744-x

**Published:** 2017-05-12

**Authors:** O. El-Atwani, J. E. Nathaniel, A. C. Leff, K. Hattar, M. L. Taheri

**Affiliations:** 1 Department of Materials Science & Engineering, Drexel University, Philadelphia, PA USA; 2 Department of Radiation Solid Interactions, Sandia National Laboratories, Albuquerque, NM USA; 3 Materials Science and Technology Division (MST-8), Los Alamos National Laboratory, Los Alamos, NM USA

## Abstract

Crystal defects generated during irradiation can result in severe changes in morphology and an overall degradation of mechanical properties in a given material. Nanomaterials have been proposed as radiation damage tolerant materials, due to the hypothesis that defect density decreases with grain size refinement due to the increase in grain boundary surface area. The lower defect density should arise from grain boundary-point defect absorption and enhancement of interstitial-vacancy annihilation. In this study, low energy helium ion irradiation on free-standing iron thin films were performed at 573 K. Interstitial loops of *a*_*0*_/2 [111] Burgers vector were directly observed as a result of the displacement damage. Loop density trends with grain size demonstrated an increase in the nanocrystalline (<100 nm) regime, but scattered behavior in the transition from the nanocrystalline to the ultra-fine regime (100–500 nm). To examine the validity of such trends, loop density and area for different grains at various irradiation doses were compared and revealed efficient defect absorption in the nanocrystalline grain size regime, but loop coalescence in the ultra-fine grain size regime. A relationship between the denuded zone formation, a measure of grain boundary absorption efficiency, grain size, grain boundary type and misorientation angle is determined.

## Introduction

Nanocrystalline metals are considered candidates for severe environmental applications which require irradiation-resistant materials^1, 2^ due to a promise of enhanced mechanical properties (strength and ductility)^3, 4^, and radiation resistance^1, 2, 5–11^ due to their high grain boundary density in comparison to their bulk counterparts^3, 4, 12^. During applications involving radiation, such as fusion and fission reactors, materials are exposed to different energetic particles with a range of doses and dose rates, which form Frenkel defect pairs (vacancies and interstitials); a high percentage of these defects will recombine leaving a small ratio of freely migrating defects. Depending on the radiation conditions, freely migrating defects can diffuse and coalesce forming larger defect clusters (loops, voids, etc.), which can alter the morphology^13, 14^, and modify the thermal^15^ and mechanical^16^ properties. Detrimental changes in the mechanical properties such as swelling, hardening and embrittlement^16^ can ultimately result in failure^17^. In addition to the microstructural changes caused by freely migrating defects, any energetic particle introduced to the sample (helium for example) can also coalesce and combine with vacancies and voids to form bubbles, which have been shown to exacerbate the unfavorable effects on the irradiated materials^13, 18–20^.

In order to extend the life time of current nuclear reactors and to develop next generation nuclear reactors, it is essential to limit these drawbacks and engineer materials that can sustain the severe radiation conditions present during reactor operation^16^. In this context, nanocrystalline materials are thought to possess advantages in terms of radiation tolerance^21^. Grain boundaries are considered to be defect (interstitials, vacancies, loops and cluster) and particle (eg. helium) sinks^22, 23^. As a result, a high density of grain boundaries in nanocrystalline metals limits the mean free path for the migrating defects and thus the density of the freely migrating defects in the matrix^24^. It is theorized that this leads to enhanced retention and prevention of defect coalescence in grain matrices. This nanoscale phenomena is expected to translate at the microscale to increased radiation doses required for the severe structural changes. Several published experimental results demonstrate lower defect density in irradiated nanocrystalline materials (metal and ceramics) when compared with coarse grained materials^25–27^. In the modeling effort of Bai *et al*.^7^, interstitials were shown to be re-emitted from the grain boundary to recombine with the slower migrating vacancies as they approach the vicinity of the grain boundary, thus contributing to increased annihilation. Therefore, it is expected that defect densities should decrease with increasing grain refinement (decreasing grain size) based on these modeling efforts. However, few studies^5, 23, 27^ on defect density trends versus grain size in nanocrystalline materials are available. In these limited studies, the scatter in defect density as a function of grain size is dominant^23, 28^ suggesting effects of grain boundary type and the overall grain boundary character (misorientation angle and grain boundary plane) on the sink efficiency. Several studies demonstrated better defect sink behavior in high angle grain boundaries (HAGB, misorientation angle ≥15°) compared to low angle grain boundaries (LAGB)^5, 29, 30^. Some other recent studies have suggested that grain boundary (and other interfaces) crystallographic character has significant effect on defect absorption^22, 31^ and segregation behavior^32, 33^ in irradiated materials. Recently, Dunn *et al*.^34^, using spatially resolved stochastic cluster dynamics (SRSCD), demonstrated strong dependence of defect accumulation near and inside a grain boundary on vacancy and interstitial binding energies to the grain boundary. These binding energies are functions of the grain boundary character. Moreover, grain boundary sink efficiency was often correlated to denuded zone formation (defect-free zone in the vicinity of the grain boundary)^22^. Since denuded zone width was shown to be proportional to the grain boundary sink efficiency^31^, a large width of an experimentally observed denuded zone near a grain boundary demonstrates a high sink efficiency of the boundary, and a grain boundary with a large denuded zone width should possess a higher sink efficiency than another grain boundary with smaller denuded zone width. However, the questions of whether or not all grain boundaries in irradiated nanocrystalline material will have denuded zones and what factors are associated with such zone formation, such as grain size, grain boundary character, and irradiation conditions (energy, species, temperature, dose, etc.) remain unanswered.

In this paper, the effect of grain size, grain boundary misorientation angle and grain boundary type on the formation of denuded zone is investigated. For this purpose, nanocrystalline free-standing iron (Fe) films were used as a model Body-Centered Cubic (BCC) material to study denuded zone formation under *in situ* irradiation in a Transmission Electron Microscope (TEM). Grain boundary absorption of defects in the nanocrystalline and ultrafine grains is discussed based on loop density and size changes as a function of irradiation dose on different grains at the same diffraction conditions. The results revealed the effect of sink strength (function of grain boundary density or grain size) of the nanocrystalline and ultrafine grains on defect absorption. Loop density is also plotted as a function of grain size to examine any possible trend. To investigate the effect of misorientation angle and grain boundary type and size on the absorption efficiency of the grain boundaries, the grain boundaries of the irradiated samples were then examined and determined to be denuded, non-denuded or partially denuded. The results were correlated with the crystallographic orientation mapping results. The results and conclusions of this study are expected to be of vital importance to the community working on understanding the behavior of nanocrystalline materials and grain boundaries under extreme irradiation conditions.

## Methods

### Preparation

Fe films were sputter deposited on NaCl substrates and then floated onto molybdenum TEM grids. Details about the sputtering target, the sputtering process, purity of the films and the TEM sample preparation method are described elsewhere^28, 35^. The films were then annealed *in situ* in a JEOL 2100 LaB_6_ TEM using a Gatan heating stage at 873 K for 600 seconds (with a ramp rate of 30 degrees per minute), while monitoring the grain growth to achieve well-defined grain boundaries without eliminating the nanocrystalline microstructure. From previous studies^28^, the average thickness of the film at the same sputter deposition conditions is approximately 100 nm and the microstructure was columnar.

### Irradiation and Characterization

The *in situ* TEM/irradiation experiment was performed in the I^3^TEM facility^36^ in the Department of Radiation Solid Interactions at Sandia National Laboratories. Films were irradiated with 10 keV He particles at a temperature of 573 K. The irradiation dose rate was 8.74 × 10^17^ ion.m^−2^.s^−1^. Three equal dose steps were performed, each of 0.93 × 10^21^ ion.m^−2^ (final dose was 2.8 × 10^21^ ion.m^−2^).

During irradiation, the sample was titled 30° relative to the electron beam resulting in the He particles having an incident angle of 60° relative to the sample normal. The corresponding projected range of the ion beam in the sample is 80 nm as calculated by the Stopping Range of Ions in Matter (SRIM) version 2013^37^. Using SRIM, the peak depth was found to be 35 nm, which implies that 10 keV He irradiation is implanted into the film. Assuming 40 eV as a displacement damage energy threshold^38^ and 100 nm film thickness, the detailed calculation with full damage cascades option in SRIM gives an average vacancy production of approximately 31 vacancies/ion. Therefore, at the peak depth of 35 nm, the vacancy production is predicted to be 0.036 (vacancies/ion.Å) and the corresponding dpa was approximately 12.

*In situ* imaging was performed in bright-field mode using a 200 keV electron beam. Before irradiation and after each dose step, Automated Crystallographic Orientation Mapping (ACOM) was performed *in situ* via NanoMEGAS ASTAR precession diffraction^39^. A spot size of 5 nm and a step size of 2 nm were used. Further characterization (TEM imaging and ACOM) on different parts of the sample was performed in a JEOL 2100 LaB_6_ TEM using 200 keV electron beam. ACOM maps had an average reliability of 67. For loop density determination (at individual steps and at the final dose), ACOM maps were used to identify grains with reasonable diffraction condition according to conventional **g.b** criteria^40^. Image J software was then used to count the defects. Specifically, several equal-sized (1,450 nm^2^) circles were drawn randomly on every grain and the loops were counted in every circle. An average was taken to find the loop density. The circle drawing tool in Image J software was used to draw circles around the perimeter of the loops (chosen randomly) and the average area was taken. To find the grain size, Image J was used to measure the perimeter of every grain and the square root was taken of the measured value. Error was taken to be equal to ±2/Area of the circle. The loop area calculation was performed on several of the same grains at different dose steps; precession diffraction data was used to confirm no change in the diffraction conditions. Defect densities as a function of grain size were measured post irradiation. The *in situ* nature of the experiment preserved the same irradiation conditions (confirmed from ACOM), and thus, permitted the detailed study of dislocation loop density and size evolution as a function of irradiation dose.

## Results

### Loop Formation and Type

**Figure 1:**
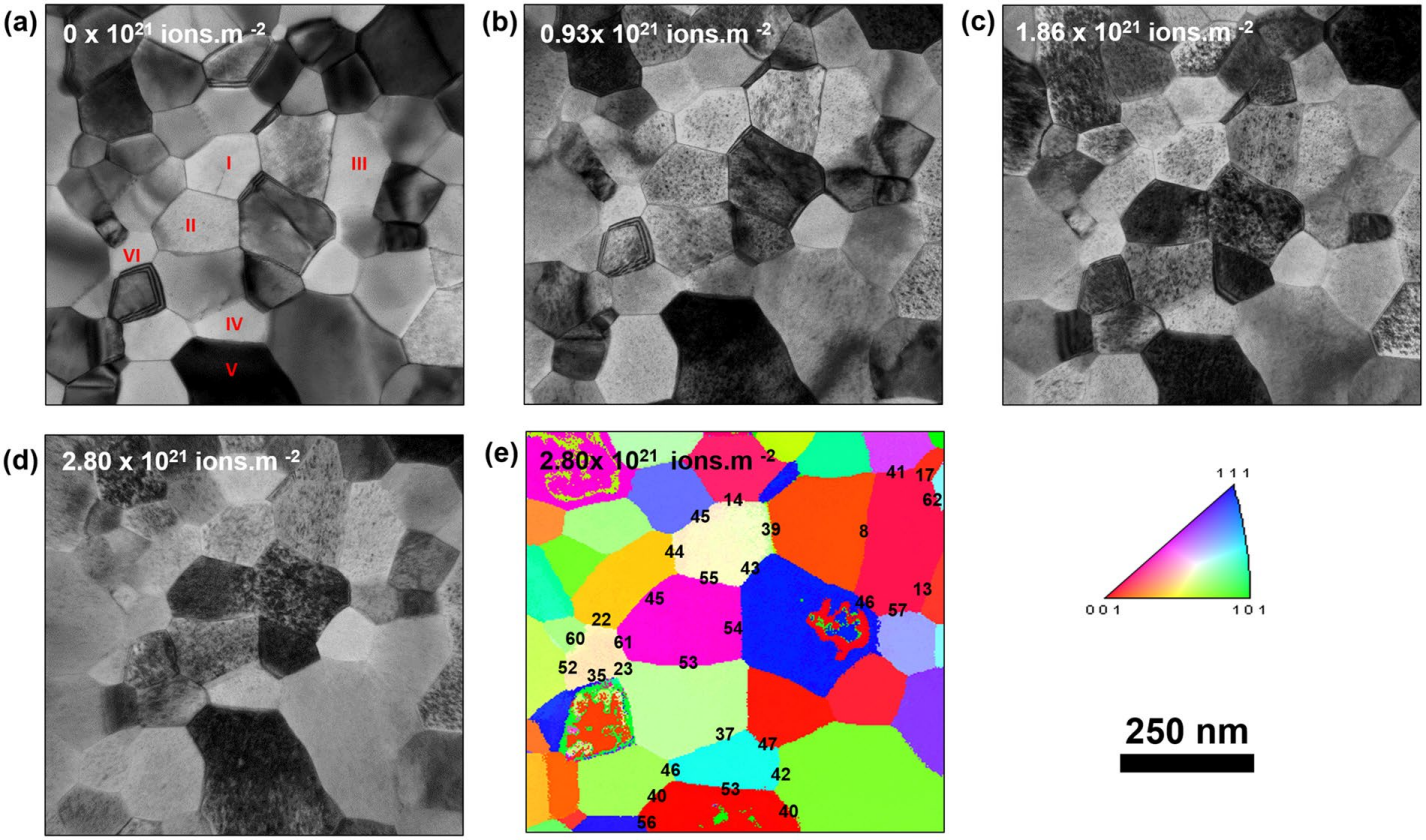
(**a**) Bright-field TEM image of free-standing nanocrystalline Fe film before irradiation. Labelled grains are used in the quantification part of loop density and size as a function of irradiation dose (**b**), (**c**) and (**d**): Bright-field TEM images of the irradiated Fe film with 10 keV He at 573 K to 0.93, 1.86 and 2.87 × 10^21^ ion.m^−2^ respectively. (**e**) ACOM map of (**d**).

Bright-field TEM images (Fig. 1) demonstrate the formation of defect loops in the nanocrystalline Fe film after being irradiated *in situ* with 10 keV helium at 573 K for three different dose steps. An Inverse Pole Figure (IPF) map of the sample at the final dose with grain boundary misorientation angles is shown in Fig. 1e. No changes in crystal orientation occurred during the course of irradiation as revealed from the IPF maps. Careful observation of the IPF maps and the corresponding bright-field TEM images demonstrated no grain growth during irradiation. To find the Burgers vector of the loops, nanobeam diffraction patterns acquired during ACOM were indexed. In irradiated BCC Fe films, it has been shown that the Burger vector is of *a*_*0*_ [100] family or *a*_*0*_/2 [111] family where the *a*_*0*_ [100] type Burgers vector form at temperatures over 673 K^28^. Seven different possibilities are then present (*a*_*0*_ [100], *a*_*0*_ [010], *a*_*0*_ [001], *a*_*0*_/2 [111], *a*_*0*_/2 [-111], *a*_*0*_/2 [1-11], *a*_*0*_/2 [11-1]). To determine the Burgers vector family in the irradiated Fe samples in this study, several **g** vectors from different grains were used. The **g.b** invisibility criteria confirmed that loops had *a*_*0*_/2<111> Burgers vectors, in agreement with previous works in irradiated Fe below 673 K^28, 41, 42^.

#### Loop Density and Area as a Function of Dose in Nanocrystalline and Ultrafine Grains

**Figure 2:**
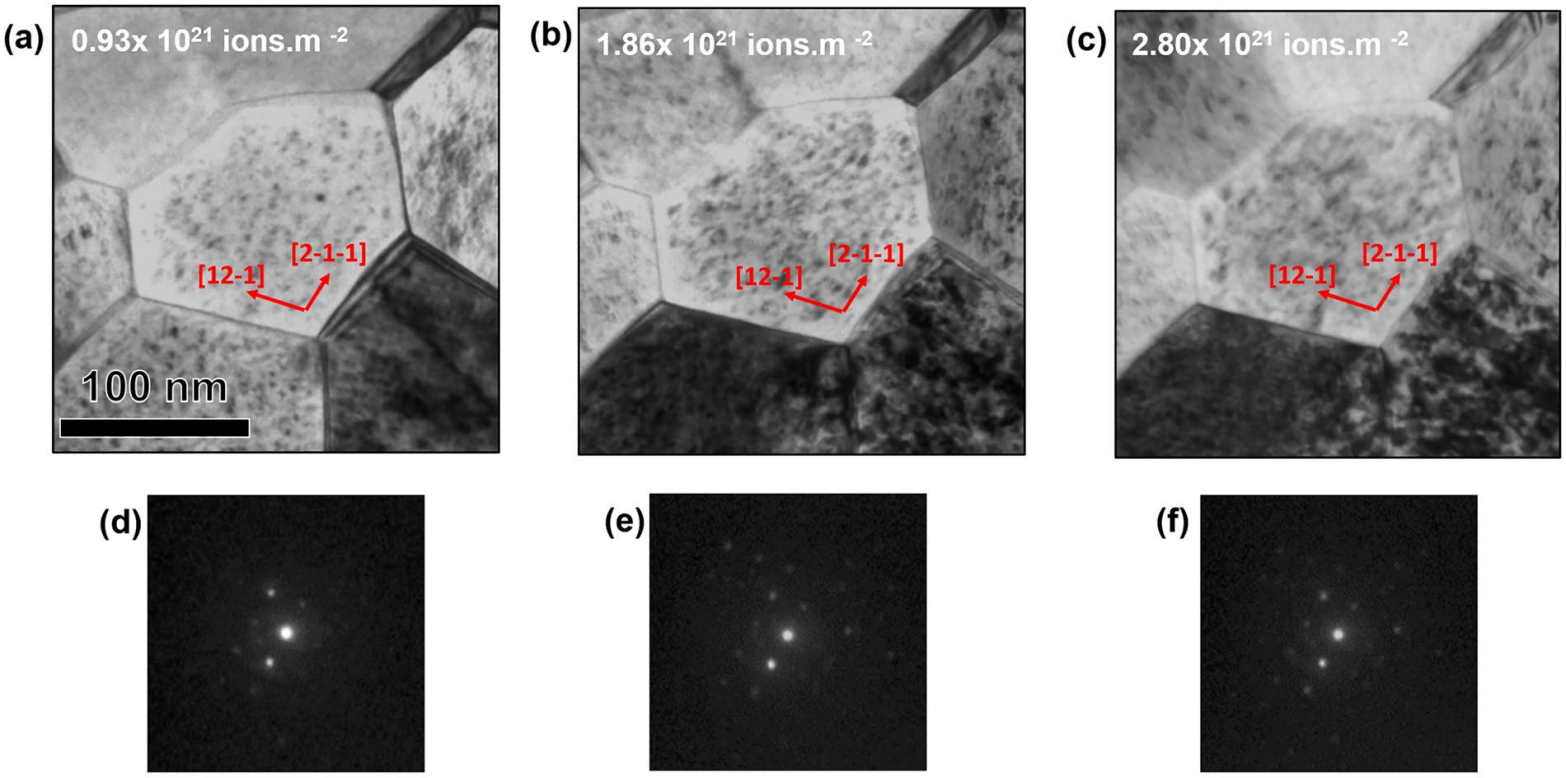
(**a**), (**b**) and (**c**) Bright-field TEM images of grain I (Fig. 1a) irradiated with 10 keV He at 573 K to 0.93, 1.86 and 2.87 × 10^21^ ion.m^−2^ respectively with g vectors of the imaging conditions. (**d**), (**e**) and (**f**) the corresponding diffraction pattern extracted from ACOM demonstrating no change in the diffraction condition during irradiation.

**Figure 3:**
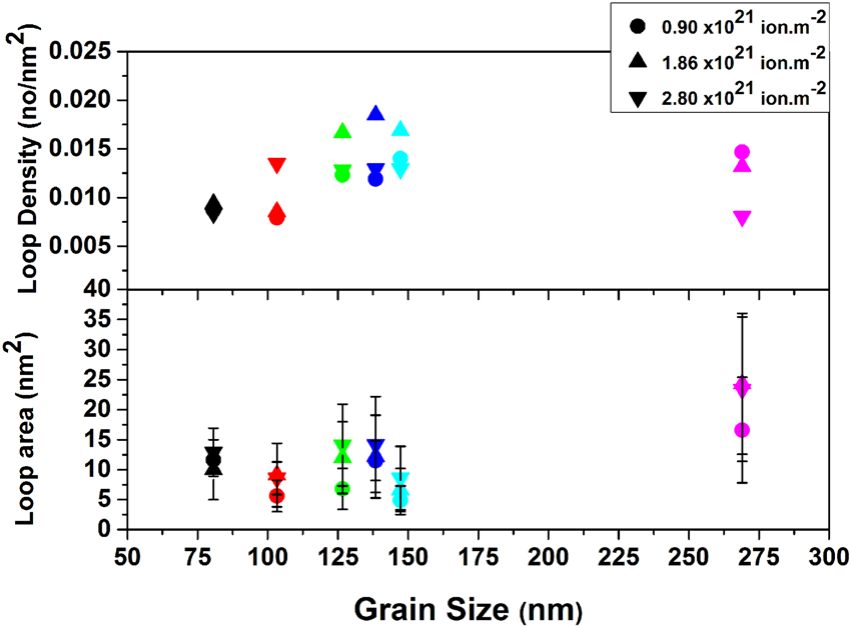
Loop areal density and loop area as a function of irradiation dose (3 irradiation steps) for grain I through VI (in Fig. 1a) as being irradiated with 10 kev He at 573 K. Diffraction conditions remained the same during irradiation. Error bars in the density graphs are smaller than the points.

Loop densities and areas were tracked in Grains 1–6 (Fig. 1), while maintaining a constant diffraction condition (Fig. 2). Keeping the same diffraction condition is a crucial aspect in the analysis since both loop density and size (contrast in the TEM image) are both dependent on **g.b**. The loop density and size as a function of irradiation dose are plotted in Fig. 3. The loop density remained nearly the same during the three irradiation steps for the very small grain (Grain VI, 80 nm diameter). Going from the first (0.93 × 10^21^ ion.m^−2^) to the second dose step (1.86 × 10^21^ ion.m^−2^), there was a slight increase in the density from 0.0087 to 0.0093 nm^−2^ and then the density decreased again to 0.0083 nm^−2^ at the last dose of 2.8 × 10^21^ ion.m^−2^). For grain IV (approximately100 nm grain diameter), the loop density increased slightly in the second irradiation step and significantly decreased during the third irradiation step. For the grains between 120–150 nm grain diameter (grains I, II, and III) the loop density increased significantly (20–33%) in the second irradiation step and then decreased significantly (23–30% during the third irradiation step). For the largest grain (Grain V, 270 nm), the loop density decreased 10 and 39% during the second and third irradiation steps, respectively. The loop area for Grain VI (80 nm) also changed little as a function of irradiation dose. Scattered behavior in terms of loop area change is noted for Grains I, II, III, and IV. A continuous decrease in area can be seen, however, for Grain V (largest grain).

To determine the loop density as a function of grain size, it is important to capture all the *a*_*0*_/2 < 111> Burgers vectors (aforementioned 4 possibilities). At particular g vectors, a fraction of these loops is invisible (**g.b** = 0). As such, a multibeam imaging condition was used to minimize this quantification error, as suggested by Jenkins *et al*.^40^. Thus, all quantified grains presented in this paper have **g** vectors to capture all the resolvable loops of the 4 possibilities *a*_*0*_/2 < 111> Burgers vectors. The loop area is determined using the diffraction contrast of the loop strain; therefore, a meaningful comparison can only be formed at the same diffracting conditions. Therefore, relative changes in size within the same grain are significant, but we cannot reliably compare between grains.

#### Loop Density vs Grain Size Trend at the Final (Maximum) Dose

**Figure 4:**
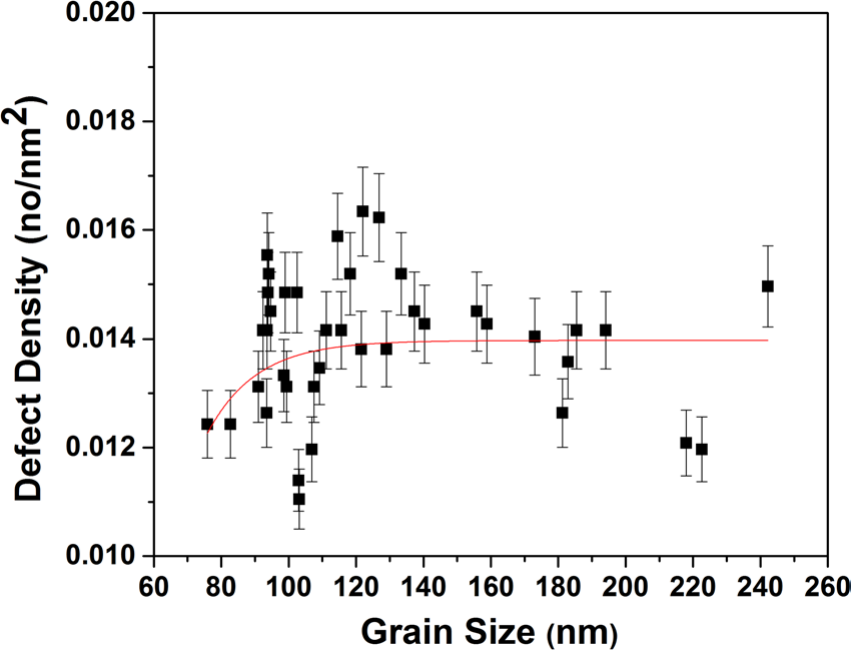
Loop areal density (loops/nm^2^) vs grain size at the end dose (2.87 × 10^21^ ion.m^−2^) of irradiated nanocrystalline Fe grains with 10 keV He at 573 K. Error bars demonstrating possible error in loop counting.

To gain additional insight into loop densities as a function of grain size, a larger sample size (total 41 grains) was analyzed from the film after the final radiation step, plotted in Fig. 4. Scattered values of the density (between 0.01 and 0.017 nm^−2^) is observed on the edge of the nanocrystalline regime (grain sizes between 90 and 120 nm). Consistent with the results at other doses, some the ultra-fine (100–500 nm grain diameter) grains tend to have low loop densities. A fitting curve demonstrated a small increasing trend in the nanocrystalline regime, which then saturated during the transition from the nanocrystalline to the ultra-fine regime.

#### Denuded Zone Dependence on Grain Boundary Character

**Figure 5:**
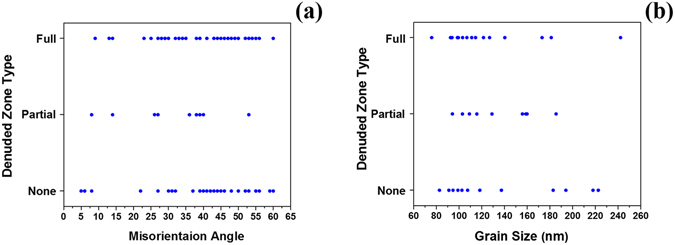
(**a**) and (**b**) Loop denuded zone type vs size grain size and misorientation angle respectively.

Correlating denuded zone formation with other factors such as grain size, type, and angle can assist in understanding the sink efficiency dependence of the grain boundaries used in this study on those parameters. The effect of grain size, grain type, and grain boundary misorientation angle on denuded zone formation are shown in Fig. 5, as generated from analyzing the bright-field TEM images and the corresponding ACOM data for the grains shown in Fig. 4. For both Fig. 5a and b, boundaries were classified into fully denuded boundaries (the majority of the boundary has an interstitial loop-free zone in the vicinity of the grain boundary), non-denuded boundaries (no interstitial loop-free zone in the vicinity of the grain boundary), and partially denuded grain boundaries (about half of the boundary has an interstitial loop-free zone in its vicinity).

**Figure 6:**
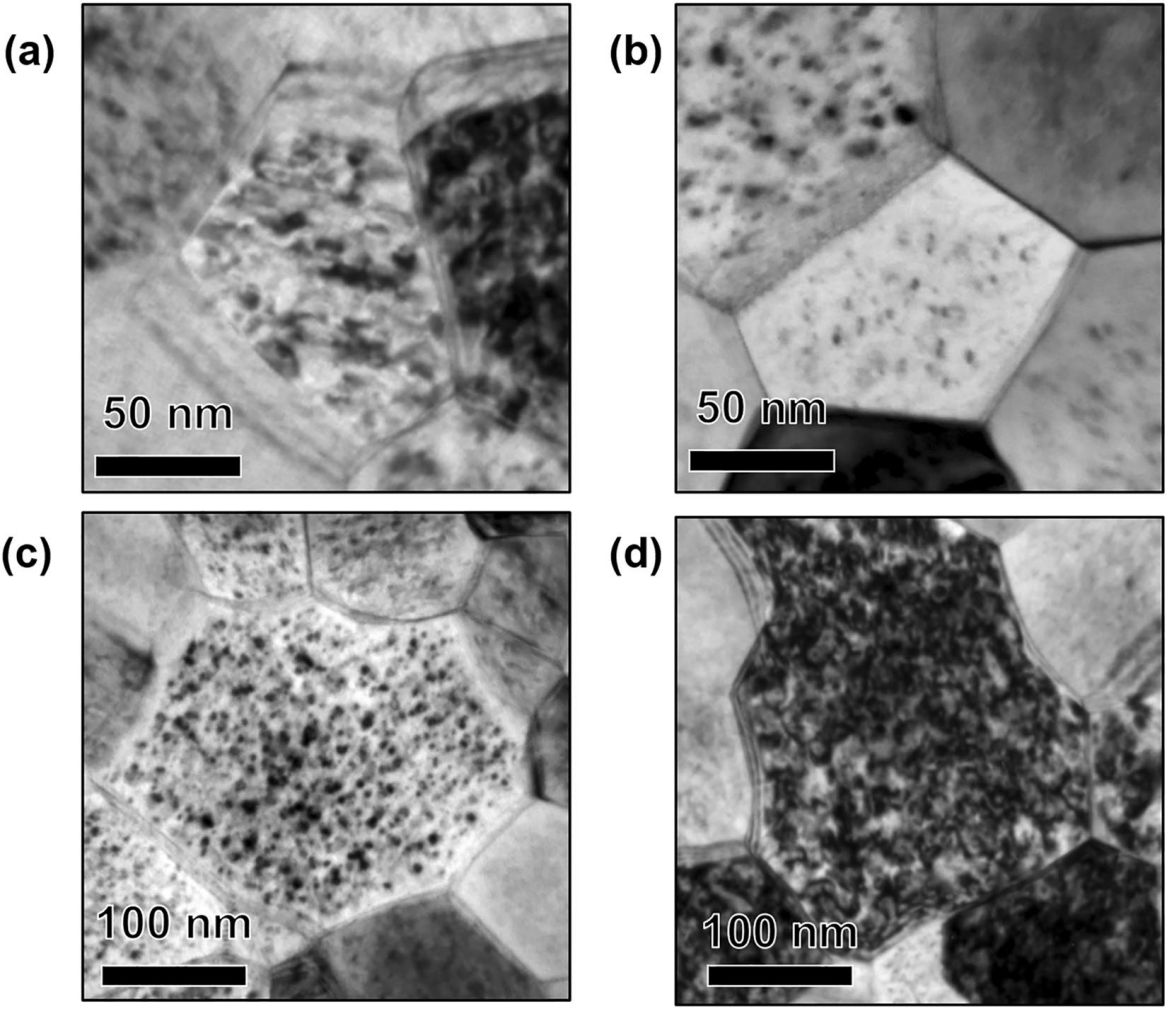
(**a**) and (**b**) Bright-field TEM image of irradiated nanocrystalline grains (approximately 80 nm average size) with loop denuded and non-denuded zones respectively. (**c**) and (**d**) Bright-field TEM image of irradiated ultra-fine grains (approximately 220 nm average size) with loop denuded and non-denuded zones respectively. Images taken at the end dose (2.87 × 10^21^ ion.m^−2^).

**Figure 7:**
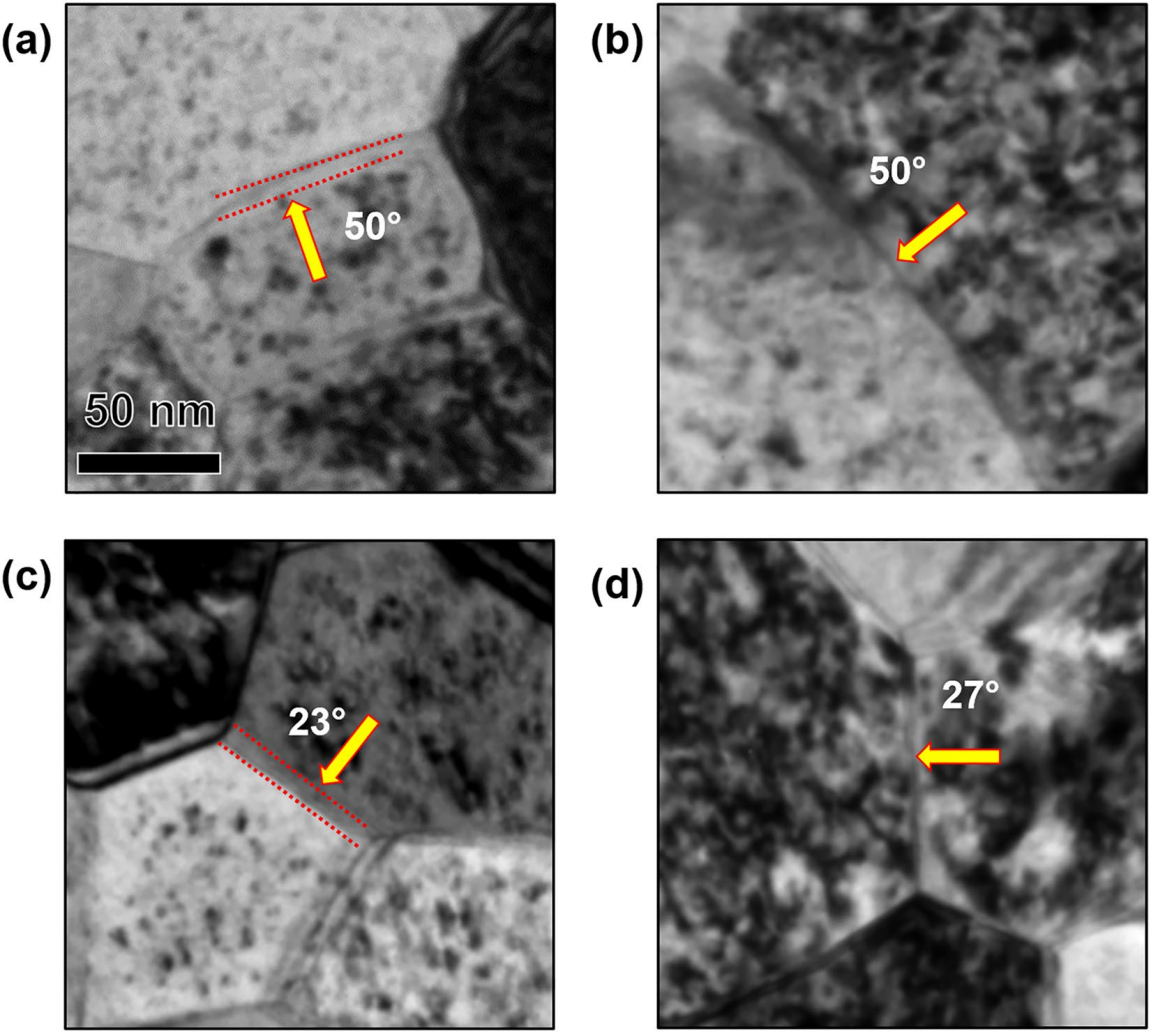
Bright-field TEM images of (**a**), (**c**) loop denuded and (**b**), (**d**) non-denuded HAGB of irradiated nanocrystalline Fe at the end dose (2.87 × 10^21^ ion.m^−2^). Misorientation angles are labelled in the Figure.

In Fig. 5, no dependence of denuded zone formation on grain size and grain boundary misorientation angle for HAGBs is readily observed. This lack of dependence is further demonstrated in the bright-field TEM images presented in Figs 6 and 7.

**Figure 8:**
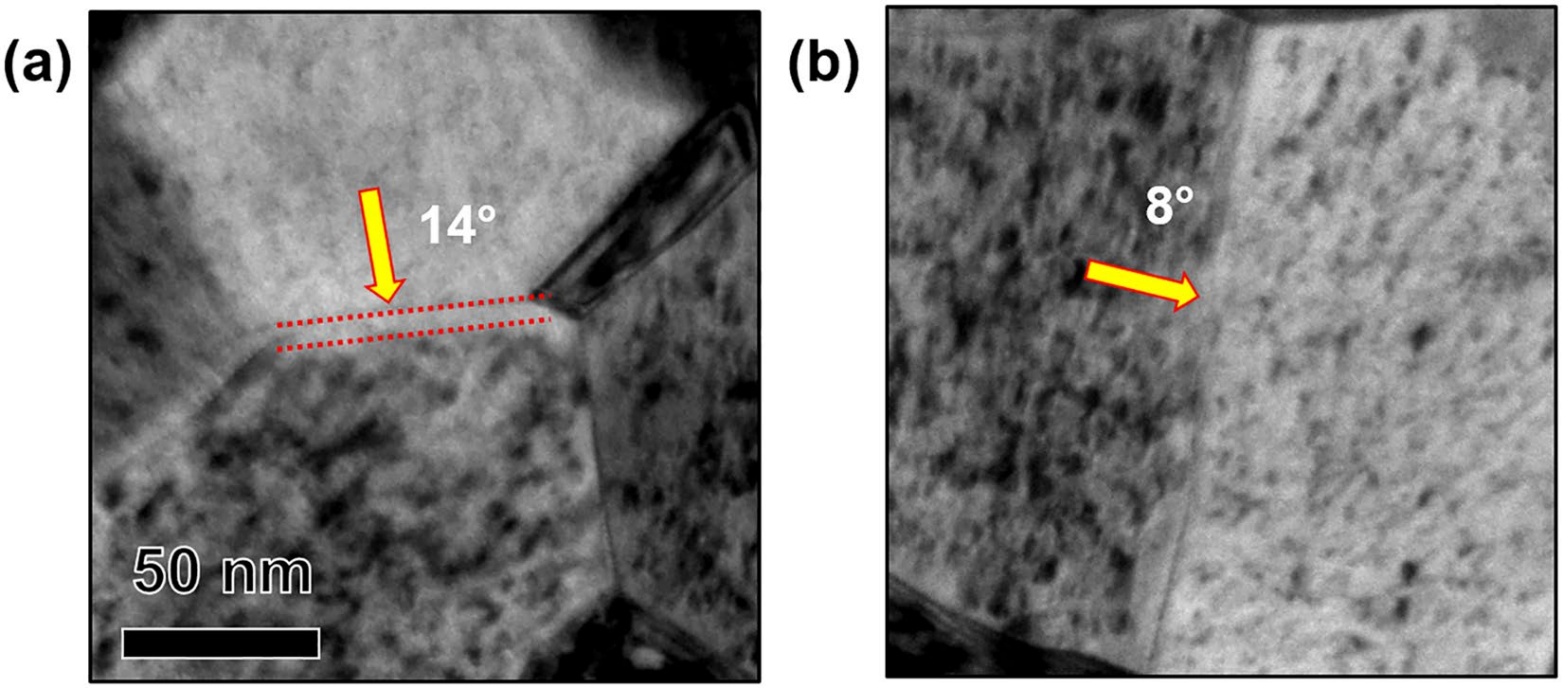
Bright-field TEM images of (**a**) loop denuded and (**b**) non-denuded LAGB of irradiated nanocrystalline Fe at the end dose (2.87 × 10^21^ ion.m^−2^). Misorientation angles are labelled in the Figure.

From Fig. 5, similar to HAGBs, it is clear that LAGBs can be denuded and non-denuded. Bright-filed images of denuded and non-denuded LAGBs are seen in Fig. 8. However, based on the small sample size of low angle boundaries, it appears that a correlation between misorientation angle and denuded zone formation for LAGBs exist (Fig. 5).

## Discussion

### Loop Density vs Grain Size and Sink Strength

The loop density vs grain size in Fig. 3 (confirmed by larger sample size in Fig. 4) demonstrated no clear trend in the transition from the nanocrystalline to the ultra-fine regime (approximately100 nm grain size). In thin films such as the one studied here (100 nm thickness), the free surfaces can also act as point defect sinks. The effect of film thickness on defect density was studied and confirmed before by Li *et al*.^43^ where defect densities were shown to decrease with decreasing the TEM foil thickness. The foil thickness effect was also dependent on the irradiation dose (increases with the irradiation dose). In addition, image forces, where glissile prismatic loops were shown to glide to free surfaces in BCC materials^44^ can also result in a decrease in defect density. To minimize these effects, all grains quantified were chosen from close regions in the film where mass thickness contrast was not observed in the TEM. It should be mentioned that surface effects are expected to play more significant role in large grains due to the longer diffusion path of the defects to the grain boundaries. Therefore, one expects the large grains to have less irradiation damage. However, the large damage observed in the large grains (represented by larger size interstitial loops and higher loop densities) confirms the enhanced performance of the nanocrystalline grains (where surface effects are less) and that the surface sink effects did not dominate in this experiment.

At the irradiation conditions in this study, both interstitial and vacancy point defects are presumed to be mobile^45^. The high density of grain boundaries is assumed to absorb migrating vacancies and interstitials. However, interstitials are expected to have faster migration and biased annihilation at the grain boundaries^46^, which should lead to less density of interstitial loops and a clear loop density versus grain size trend.

Previous studies demonstrated either scattering behavior or even an opposite trend (defect density decreases with grain size)^23, 28^. Comparing the loop density, an area of the selected grains in Fig. 1 assist in understanding why no clear trend is observed especially during the transition from the nanocrystalline regime to the ultra-fine regime. After nucleation, loops can grow with loss of interstitials to the loops according to the following equation^47^:

1 $$\frac{d{q}_{i}^{IL}}{dt}={k}_{i}^{{2}^{IL}}{D}_{i}{C}_{i}-\,{k}_{v}^{{2}^{IL}}{D}_{v}{C}_{v}+{K}_{v}^{IL}$$

where ${q}_{i}^{IL}$ is the fraction of interstitials being lost to interstitial loops, ${k}^{{2}^{IL}}$ is the sink strength of the interstitial loops to point defects, *D* and *C* are the diffusion coefficients and the concentration of point defects, i denotes interstitials and v denotes vacancies. The final term ${K}_{v}^{IL}$ is the rate of vacancy emission from such loops.

Focusing on grain VI (nanocrystalline grain with approximately 80 nm in diameter), despite continuous formation of interstitial defects during irradiation and the enhancement of loop formation via bubble formation (though loop punching and trap-mutation processes)^48^, the loop density and size (Fig. 3) showed little changes compared to other grains. This suggests a decrease in the first term in equation [1] due to strong interstitial absorption by grain boundaries.

On the other extreme, focusing on grain V (ultrafine grain with approximately 270 nm grain diameter), the loop density showed a significant decrease, while the loop area demonstrated significant increase. The decrease in the loop density in this case (ultra-fine grain) suggests the occurrence of loop coalescence. This is demonstrated by the large differences in loop areas in the grain as indicated by the large standard deviation in Fig. 3. Grains I, II, III and IV (ultra-fine grains), however, showed an increase in defect density during the second irradiation step and a significant decrease in loop density during the third irradiation step. The loop area in these grains, however, showed an increase in the second irradiation step but a little change in the third irradiation step. This suggests that the grain boundaries in these grains were less efficient at interstitial absorption than Grain VI in the second irradiation step (loop area was increasing together with loop density) and that grain boundary-loop absorption occurred during the third irradiation step (loop density was decreasing but loop area showed little change). Grain boundary strength in defect absorption is then decreasing as the grain size increases (going from the nanocrystalline to the ultrafine regime). However, the behavior of different grain sizes regarding defect absorption and loop coalescence during irradiation should disrupt any trend expected as the grain size increases from the nanocrystalline regime to the ultra-fine regime. For example, a large grain can show less loop density than a nanocrystalline grain, but much larger loops due to loop coalescence and as a result contain more displacement damage. Loop coalescence in irradiated nanocrystalline and coarse grained materials^23, 49, 50^, as well single crystalline materials were previously observed^51^. At high doses, loop coalescence can lead to a scattering or opposite trend as observed in previous studies^23, 28^. Therefore, determining the loop density and size as a function of irradiation dose on nanocrystalline and ultrafine grains at the same diffraction conditions (same **g.b** contrast) revealed the effect of grain size on defect absorption and confirmed the role of loop coalescence in disrupting loop density trend as a function of grain size.

The loop density change as a function of grain size for a large sample size (high number of quantified grains) is demonstrated in Fig. 4. The scattered behavior in the nanocrystalline regime (grain size of 90–140 nm) can be a result of other factors such as ion channeling, grain boundary misorientation angle, type of boundary (HAGB vs LAGB) or crystallography (character) differences which will be discussed below. The difference in surface effects (due to proximity to the surface) is a minor factor since these grains have similar sizes. Moreover, a cut-off grain size in the nanocrystalline regime below which efficient grain boundary defect absorption and significant decrease in loop or bubble density occur, has been reported on another BCC nanocrystalline material^9^. The cut-off value was around 50–60 nm^9^, below the grain sizes used in this study which may explain the non-clear trend observed in the edge of the nanocrystalline regime (approximately 80–100 nm) if a similar cut-off value is valid on nanocrystalline Fe.

### Denuded Zone Formation and Sink Efficiency

A low defect density in a specific grain compared to other grains in the nanocrystalline regime is related to the efficiency of its grain boundaries in annihilating point defects and/or absorb defect clusters. The efficiency of the grain boundary in capturing point defects, however, is correlated with denuded zone formation near the boundary^22^. Following the argument by Beyerlein *et al*.^31^, void denuded zones and its dependence on sink efficiency, equation 2.1.6 in ref. 31 can be written in a form in which the vacancy parameters are replaced by interstitial parameters as follows:

2 $$\,{\lambda }_{DZ}\sqrt{\frac{{K}_{si}}{{D}_{v}}}=\,\mathrm{ln}\,{{\rm{\eta }}}_{i}-\,\mathrm{ln}(1-{\rm{\Delta }}{c}_{i}\frac{{k}_{si}}{{k}_{0}})$$

where *λ*_*DZ*_ is the width of the denuded zone, *K*_*si*_ is the interstitial-sink reaction rate coefficient, *k*_0_ is defect production rate, *D*_*i*_ is interstitial diffusivity, *c*_i_ is the interstitial concentration and η_*i*_ is the interstitial sink efficiency. η_*i*_ is ratio of interstitial flux going into the sink in interest to the interstitial flux going into a perfect sink and, therefore, can range from 0 (no vacancy absorption) to 1 (perfect sink). From the equation, denuded zone size is directly proportional to sink efficiency.

In this work, the independence of denuded zone formation on misorientation angle of HAGBs is in agreement with results of Tschopp *et al*.^30^ on grain boundaries of α-Fe. According to that study, the vacancy and interstitial formation energies (thus, sink efficiency) on grain boundary sites demonstrated very little correlation with grain boundary misorientation angle. Tschopp *et al*.^30^, however, found a correlation between misorientation angle in LAGBs and vacancy and interstitial formation energy in agreement with our work (Fig. 5). The results, however, do not rule out the dependence of sink efficiency on misorientation angle of the boundary, since the grain boundary habit plane also influences grain boundary sink efficiency, and is another factor to consider. Mobility of defects in the boundary plane dictates the potential for vacancy-interstitial recombination and, thus, affects its efficiency in absorbing defects^31^. If denuded zone formation, which needs more detailed investigation and better understanding, marks the sink efficiency of a grain boundary, our results then confirms the dependence of sink efficiency on grain boundary character. However, since grain boundaries with same misorientation angle can be denuded and non-denuded (Fig. 5), and no trend in denuded zone width exist as a function of misorientation angle, our results demonstrates larger dependence of grain boundary sink efficiency on the grain boundary plane.

In this study, denuded zone formation was also investigated in coincidence site lattice (CSL) boundaries particularly ∑3[111] boundaries. Out of 27 grain boundaries analyzed, only two boundaries demonstrated denuded zone formation. Based on ∑3[111] type grain boundary energies^30^, ∑3 {112} [111] (a coherent boundary) has the lowest formation energy and is most common. Vacancy and interstitial formation energies were found to be 1.53 and 3.15 eV respectively on ∑3 {112} [111] which are very comparable to their bulk formation energies (3.52 and 1.72 respectively)^30^. Therefore, these coherent boundaries have less effect in defect annihilation enhancement and no denuded zone formation is expected^52, 53^.

More careful examination of denuded zone formation and possible depletion is necessary to understand the correlation of denuded zone formation and grain boundary sink efficiency. Possible interstitial loop diffusion towards boundaries, the interaction (absorption) of small loops with grain boundaries, and the possible dependence of grain boundary sink efficiency on time should be well understood before a final conclusion regarding denuded zone formation and its correlation with sink efficiency can be determined.

### Channeling Effect

**Figure 9:**
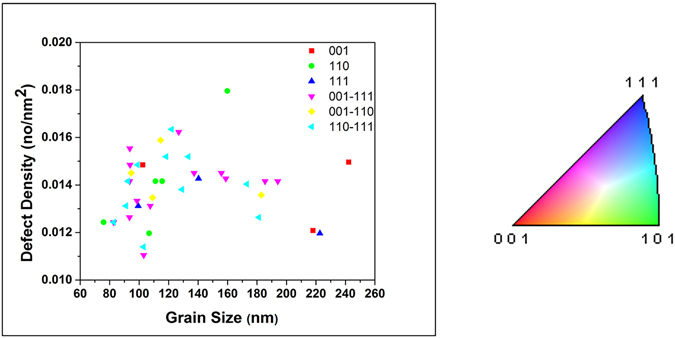
Loop areal density (no/nm^2^) vs grain size (area) showing the orientations of the grains (6 different orientations extracted from the ACOM maps) of irradiated nanocrystalline Fe grains. Colors are taken to match those in the polar image (right) taken from the ACOM maps.

Of the important factors to consider in He irradiation of Fe is the channeling effect. Channeling ions create less defects which should affect interstitial loop formation and density. Ion channeling has been described and applied in several studies^52–54^. Kempshall *et al*.^54^. Channeling was ruled out in this experiment, however, due to a lack of correlation between grain orientation and loop density (Fig. 9). It should be mentioned, however, that the irradiation was performed at 60° incidence and post-irradiation ACOM was performed using a beam rotating around a spot the specimen normal, and therefore, variations can occur in grain orientations when tilting. However, grains near [001] orientations should show limited variations and can be used to support our conclusion.

### Bubble Effect on Defect Density Trend

Bubble nucleation and helium-vacancy complex formation^45^ due to helium implantation are present and should affect loop formation through some synergistic effects. Helium-vacancy complexes He_n_V_m_, which form in picoseconds during irradiation^55^ with high n/m ration are stable and can grow by absorbing vacancies to form bubbles. Stewart *et al*.^56^ has shown that coalescence of mobile helium atoms punch out interstitial atoms leaving out stable helium-vacancy complexes, which are immobile at temperatures below 573 K^57^ (the temperature in this study). These stable helium-vacancy complexes were shown to trap interstitial defects in their vicinity and aid the nucleation of interstitial loops^58^. Bubbles, on the other hand, can punch out interstitial loops (a process usually referred as loop punching)^48^. In the molecular dynamics calculation study by Lucas and Schaublin^59^, helium was shown to favor the formation of interstitials in the displacement cascades, while the dual beam *in situ* TEM study of Brimbal *et al*.^50^ demonstrated decreased interstitial loop mobility in the presence of helium when compared to pure Fe irradiation.

Studies^50, 58^ confirmed that helium-vacancy complexes lead to higher loop densities. Therefore, bubble density is an important factor to consider when discussing loop density as a function of grain size in helium irradiated samples. Bubbles, however, showed a uniform distribution among the grain and the grain boundaries since helium-vacancy complexes are mobile at temperatures higher than 573 K and therefore, they have limited effect on the loop density vs grain size trend determined in this study.

## Conclusion

The results in this *in situ* TEM/irradiation study on free standing nanocrystalline iron films demonstrated several significant findings about the behavior of nanocrystalline materials under irradiation. These findings provide fundamental aspects to be considered in engineering radiation tolerant nanomaterials, and are summarized below:

- The 10 keV He irradiation on nanocrystalline iron at 573 K resulted in the formation of dislocation loops with *a*_*0*_/2 <111> Burgers vectors.
- The overall loop density vs grain size demonstrated a slow increasing trend in the nanocrystalline regime, scattering in the edge of the nanocrystalline regime as well as in the transition from the nanocrystalline to the ultra-fine regime.
- The occurrence of loop coalescence was confirmed by comparing loop density and loop area during irradiation as a function of dose.
- Several factors that can affect the trend in defect density versus grain size and cause scattering behavior were studied. First, channeling which can occur in BCC materials and is strongest in the highest density direction (<111>) was ruled out to be a dominant factor in effecting the loop density trend. Grains with similar orientations and grain size are demonstrated to have different loop densities. In addition, no correlation was observed between grain orientation and the loop density trend.
- Since grain boundary efficiency, the factor affecting the loop densities in the nanocrystalline grains, has often been correlated to denuded zone formation, denuded zone formation was studied as a function of grain size, misorientation angle and type of the boundaries. Denuded zone formation was shown to be independent of grain size and misorientation angle in the HAGBs. A correlation was demonstrated to occur between misorientation angle and denuded zone formation near LAGBs, however.

Several outstanding questions are yet to be answered for better understanding of the irradiation resistance of nanocrystalline materials. Studies regarding the effect of irradiation parameters (e.g. temperature^60, 61^) on denuded zone formation and sink efficiency should assist in understanding the material behavior under irradiation The irradiation resistance of nanocrystalline materials can be specific to the irradiation conditions. Some materials can be more resistant to irradiation than others at similar irradiation conditions due to the differences in material response (e.g. defect mobilities). Therefore, systematic studies are to be performed on different materials at different conditions before generalizing any correlation between the overall character of the grain boundary (sink efficiency) and the performance of nanocrystalline materials. Synergistic effects between the different factors such as helium ion interactions with the vacancies (in helium irradiated materials) and the effect of helium-vacancy complexes on defect annihilation and interactions with the grain boundaries should be well understood. In addition, grain boundary-loop interaction, denuded zone formation mechanism and their possible collapse at high irradiation doses are to be studied to better understand the performance of nanocrystalline materials under irradiation.
